# Human Parechovirus Infections in Canada

**DOI:** 10.3201/eid1206.051675

**Published:** 2006-06

**Authors:** Yacine Abed, Guy Boivin

**Affiliations:** * Centre Hospitalier Universitaire de Québec, Quebec City, Quebec, Canada;; † Laval University, Quebec City, Quebec, Canada

**Keywords:** Human parechoviruses, picornaviruses, RT-PCR, phylogenetic tree, pediatric infections, hospitalization

## Abstract

A new reverse transcription–polymerase chain reaction assay was developed for identification of 28 Canadian human parechovirus (HPeV) isolates, including 20 HPeV-1, 3 HPeV-2, and 5 HPeV-3, recovered from 1985 to 2004. All HPeV-1 isolates but 1 were genetically distinct from the Harris reference strain. One HPeV-2 isolate was related to the Williamson strain; the other 2 were related to the Connecticut strain. HPeV-3 isolates clustered together. Seventy-five percent of isolates were recovered during the typical enterovirus season. All patients but 1 were children with a mean age of 14.6 months, 6.3 months, and 0.7 months for HPeV-1, HPeV-2, and HPeV-3 patients, respectively. All HPeV-2– and HPeV-3–infected children were hospitalized with a diagnosis of viremia or sepsis. HPeV-1–infected children had bronchiolitis diagnosed in 50% of the cases, with few cases of pneumonia and enteritis. Two infected patients (1 child with leukemia and a 78-year-old woman) died of septic shock and severe pneumonia, respectively.

## Abstract 2

These infections are associated with a variety of clinical syndromes, in part related to specific serotype.

Picornaviruses constitute a diverse family of single-stranded positive-sense RNA viruses whose genome is packed into a nonenveloped icosahedral capsid (1). Within the *Picornaviridae* family, 5 genera are known to cause infections in humans: *Enterovirus*, *Hepatovirus*, *Rhinovirus*, *Kobuvirus*, and *Parechovirus* (1). The *Parechovirus* genus contains 3 human pathogens (HPeV-1, -2, and -3) as well as Ljungan virus, a rodent parechovirus isolated from bank voles (2). These viruses have several atypical biologic and molecular properties compared to other picornaviruses, such as unusual cytopathic effects and the lack of cleavage of the VP0 protein into VP4 and VP2, which results in a virion particle with only 3 capsid proteins rather than 4 (3).

Previous studies demonstrated that HPeV-1 (formerly echovirus 22) had a worldwide distribution and was associated with diseases similar to those caused by human enteroviruses (HEVs), i.e., gastroenteritis, respiratory diseases, aseptic meningitis, encephalitis, and neonatal sepsis–like syndromes (3–6). In general, HPeV-1 seems to be responsible for more gastrointestinal and respiratory syndromes and for fewer central nervous system (CNS) symptoms than enteroviruses (3,6). HPeV-2 (formerly echovirus 23) has been rarely reported, despite its early description in 1961 (7). In a retrospective Swedish study covering a period of >30 years, only 5 cases of HPeV-2 infections (including 4 cases of gastrointestinal symptoms and 1 case of respiratory symptoms) were reported (8), compared to 109 HPeV-1 infections during the same period (9). The third type of HPeV (HPeV-3) was reported in 2004 from stool specimen of a 1-year-old Japanese girl with transient paralysis (10). Subsequently, we reported 3 cases of sepsislike illnesses attributable to HPeV-3 infections in Canadian neonates (11). The association of HPeV-3 with 3 cases of sudden infant death syndrome was also suggested (12). In a recent Dutch study, HPeV-3 was shown to be more involved in CNS infections than HPeV-1 (13).

Similar to HEVs, HPeVs infections are commonly identified by virus isolation in cell culture, followed by neutralization typing (6,9). Isolation in cell culture is laborious and time-consuming. In addition, the method may lack sensitivity, thus leading to false-negative results (14,15). Because of increasing knowledge of genome sequences of HPeVs, development of molecular techniques such as reverse transcription–polymerase chain reaction (RT-PCR) could be an important alternative tool for specific and sensitive detection of these viruses. The increased sensitivity of RT-PCR compared to virus isolation has already been demonstrated for other clinically important picornaviruses such as enteroviruses and rhinoviruses (16–19). Nevertheless, HPeVs could not be detected by most molecular assays designed for HEV diagnosis because of considerable sequence differences between these 2 viral genera (20–22). In this study, we developed a single RT-PCR test for detecting the 3 HPeV types based on sequence alignments of HPeV-1, -2, and -3 reference strains and various clinical HPeV isolates. This test was applied for the identification of several putative HPeV isolates recovered in Quebec (Canada) during the last 2 decades (1985–2004).

## Materials and Methods

### Viral Strains and Cell Culture Procedures

A total of 30 HPeV strains, including the reference strains Harris (HPeV-1, ATCC VR-52), and Williamson (HPeV-2, ATCC VR-53), as well as 28 clinical HPeV isolates were used in this study (Table). Clinical specimens of various origins, such as nasopharyngeal aspirates (NPA), throat swabs, stools, cerebrospinal fluid (CSF), and endotracheal secretions (Table A1), were first added to different continuous cell lines, including human lung adenocarcinoma (A-549), human rhabdosarcoma (RD), transformed human kidney (293), human colon adenocarcinoma (HT-29), human laryngeal carcinoma (Hep-2), human foreskin fibroblast, mink lung, African green monkey kidney (Vero), Madin Darby canine kidney , and rhesus monkey kidney (LLC-MK2) cells. The viral cultures were incubated for 3 weeks at 37°C in a 5% CO_2_ atmosphere. Viral isolates with cytopathic effects (CPE) suggestive of HEV or HPeV were further analyzed by neutralization assays with Lim and Benyesh-Melnick antiserum pools A-H (National Institutes of Health, Bethesda, MD, USA) and specific antisera for HPeV-1 and -2 (MA Bioproducts, Walkersville, MD, USA).

**Table:** Molecular and virologic characteristics of Canadian HPeV isolates*

Virus	HPeV type	% nt/aa identities†	Neutralization assay‡
Harris	1	NA	+
Williamson	2	NA	+
Can4541-85	1	76.7/89.9	+
Can-5188-85	1	76.8/89.9	+
Can11750-87	1	76.8/89.9	+
Can11758-87	1	76.8/89.9	+
Can29218-90	1	76.4/89.2	+
Can29192-90	1	75.0/89.6	+
Can40057-93	1	76.8/89.6	+
Can41934-93	1	77.1/89.6	+
Can61165-97	1	77.0/89.9	+
Can82753-01	1	76.5/89.2	+
Can81805-01	1	85.1/97.5	+
Can87376-02	1	76.4/89.6	+
Can87639-02	1	76.4/89.6	+
Can88461-02	1	77.1/89.9	+
Can88770-02	1	76.3/89.2	+
Can85372-02	1	76.5/89.2	+
Can84436-02	1	76.9/89.6	+
Can101909-04	1	76.6/89.6	+
Can102318-04	1	76.6/89.6	+
Can100121-04	1	77.0/89.2	+
Can82047-01	2	81.1/96.4	+
Can95219-03	2	67.8/72.5	–
Can95224-03	2	67.8/72.5	–
Can81235-01	3	95.3/96.1	–
Can81554-01	3	95.0/95.7	–
Can82853-01	3	95.5/96.4	–
Can99190-04	3	95.0/96.1	–
Can97858-04	3	95.0/96.4	–

*HPeV, human parechovirus; nt, nucleotide; aa, amino acid; NA, not applicable. †% identities were calculated by comparison with Harris (accession no. S45208), Williamson (accession no. AJ005695) and A308/99 (accession no. AB084913) reference strains for HPeV-1, -2 and -3 isolates, respectively. ‡Neutralization assays were performed by using specific HPeV-1 and -2 (formerly echovirus 22 and 23, respectively) antisera (MA Bioproducts, Walkersville, MD, USA).

### Molecular Characterization

HPeV genomic RNA was isolated from the supernatant of infected cell cultures with the QIAamp Viral RNA kit (Qiagen, Mississauga, Ontario, Canada). cDNA was prepared by using the HPeVUniv5´ primer selected from the C-terminal region of the capsid VP0 encoding gene: 5´-GCT GAC CTA TGY ATC CCC TAT GT-3´ (nt 1358–1379, GenBank accession no. AJ998818) and the SuperScript II reverse transcriptase (Gibco BRL, Burlington, Ontario, Canada). Viral cDNA was then amplified by PCR by using the *Pfu* Turbo Polymerase (Stratagene, La Jolla, CA, USA) and primers HPeVUniv5´ and HPeVUniv3´ (selected from the N-terminal region of the capsid VP1 encoding gene): 5´-GTG AAC CCC AYG AAT TTT GGA A-3´ (nt 2351–2330, accession no. AJ998818). After a primary denaturation step at 95°C for 5 min, 35 cycles of denaturation at 94°C for 50 s, annealing at 58°C for 50 s, and extension at 72°C for 2 min were performed, followed by a final extension step at 72°C for 7 min. PCR products were analyzed by electrophoresis on a 1.2 % agarose gel and the expected amplicons of ≈1,000 bp were purified by using the GenElute PCR clean-up kit (Sigma-Aldrich, St. Louis, MO, USA). Nucleotide sequences of PCR products were determined with PCR primers by using an automated DNA sequencer (ABI Prism 377A; Perkin-Elmer Applied Biosystems, Foster City, CA, USA). The nucleotide sequences were analyzed, and the deduced amino acid sequences were aligned by using ClustalW (available from http://www.infobiogen.fr/). The resulted alignment was analyzed by the neighbor-joining method with the MEGA2 (Biodesign Institute, Arizona State University, Phoenix, AZ, USA) program for the construction of the phylogenetic tree.

### Clinical Data and Statistical Analysis

Demographic and clinical informations were retrospectively collected from clinical charts of hospitalized patients for whom a positive culture for HPeV was obtained in our laboratory in the past 20 years. The Mann-Whitney rank sum test was used to compare the mean age of patients infected with HPeV-1 versus HPeV-3; the Fisher exact test was used to compare HPeV-1 versus the 2 other HPeV types regarding the rate of underlying diseases and the nature of initial diagnosis.

## Results

### Virologic and Molecular Findings

**Figure 1:**
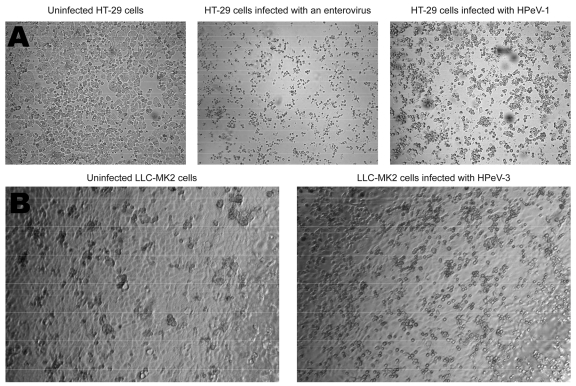
Cytopathic effects (CPEs) observed 3 days after infection of HT-29 cells with an enterovirus or a human parechovirus (HPeV)-1 isolate after 1 passage on this cell line (A); CPE observed 1 day after infection of LLC-MK2 cells with a HPeV-3 isolate after 3 passages on this cell line (B).

Among the different cell lines used, HT-29 was found to be the most suitable for efficient isolation of HPeV-1 and -2. Both grew efficiently in this cell line with an evident CPE noted after a mean incubation time of 3.8 days (range 1–8 days). A presumptive distinction between HPeV- and HEV-induced CPE in this cell line was possible. Parechovirus-infected cells were large, regularly shaped spheres, whereas enterovirus-infected cells were rather small with an irregular shape (Figure 1A). On the other hand, HPeV-3 isolates only initially grew on LLC-MK2 cells after a mean incubation time of 16 days (range 14–17 days). On passage, however, these viruses grew rapidly (in ≈1–3 days) in the same cell line (Figure 1B) and in Vero cells. All tested HPeV-1 and only one third of HPeV-2 (Can82047-01) isolates were neutralized by specific antisera for HPEV-1 and -2 (Table).

All HPeV clinical isolates as well as the 2 HPeV-1 and -2 reference strains were efficiently amplified by our RT-PCR test, generating an amplicon of the expected size (≈1,000 bp). No PCR products were generated from enterovirus (coxsackievirus A4 and coxsackievirus B4) and rhinovirus (n = 2) clinical isolates. Conversely, our HPeV isolates were negative when tested by our HEV RT-PCR assay, which is based on the amplification of the 5´ noncoding region. Sequencing of HPeV PCR products confirmed the presence of the VP3 gene in our HPeV-1 (n = 20), HPeV-2 (n = 3) and HPeV-3 (n = 5) clinical isolates. Sequence analysis showed that HPeV-1 clinical isolates had 75.0% to 85.1% nt and 89.2 to 97.5% amino acid identities with the prototype Harris strain (accession no. S45208) (Table). HPeV-2 isolates had nucleotide and amino acid identities of 67.8% to 81.1% and 72.5% to 96.4%, respectively, with the prototype Williamson strain (accession no. AJ005695). Of note, Can95219-03 and Can95224-03 HPeV-2 isolates had the same sequence and were more related to the Connecticut CT-80-6760 HPeV-2 strain (accession no. AF055846) with which they had nucleotide and amino acid identities of 85.1% and 95.8%, respectively. HPeV-3 isolates had nucleotide and amino acid identities of 95.0% to 95.5% and 95.7% to 96.4%, respectively, with the Japanese A308/99 HPeV-3 isolate (accession no. AB084913).

**Figure 2:**
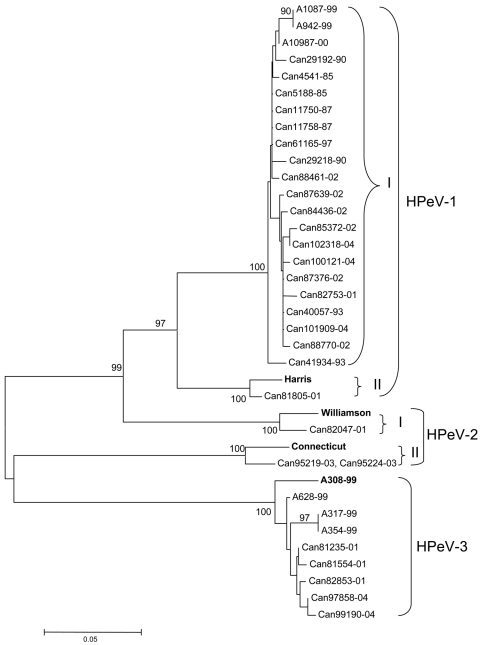
Phylogenetic analysis of Canadian human parechovirus (HPeV) isolates and HPeV-1 (Harris, GenBank accession no. S45208), HPeV-2 (Williamson, AJ005695, and Connecticut, AF055846), and HPeV-3 (A308-99, AB084913) reference strains based on the ClustalW alignment of the VP3 amino acid sequences. Japanese HPeV-1 (A1087-99, accession no. AB112485; A942-99, AB112486; and A10987-00, AB112487) and HPeV-3 (A628-99, accession no. AB112484; A317-99, AB112482, and A354-99, AB112483) isolates were also included in the analysis. The tree was constructed by using the neighbor-joining method with the MEGA 2 program. Bootstrap probabilities for 550 replicas are shown at the branch nodes. Only values of 70% to 100% are indicated. **Boldface** indicates HPeV reference strains.

Phylogenetic analysis confirmed the identities of HPeV isolates. The HPeV-1 isolate Can81805-01 was closely related to the reference Harris strain (lineage II), whereas the remaining 19 isolates from this study, as well as 3 other Japanese HPeV-1 isolates (A1087-99, A942-99, and A10987-00), were found to form a distinct cluster (lineage I) (Figure 2). The HPeV-2 isolate Can82047-01 was related to the reference Williamson strain (lineage I), whereas the other 2 HPeV-2 isolates, Can95219-01 and Can95224-01 HPeV-2, were somewhat related to the Connecticut CT80-6760 strain (lineage II). Finally, all HPeV-3 isolates formed a separate cluster that also included other Japanese HPeV-3 isolates (A308-99, A628-99, A317-99, and A354-99).

**Figure 3:**
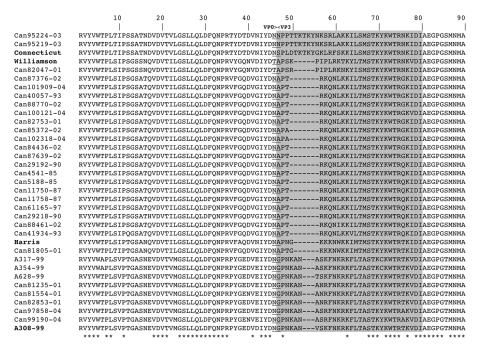
Comparison of the predicted VP0-VP3 capsid protein region of Canadian isolates with that of Harris (human parechovirus [HPeV]-1), Williamson and Connecticut (HPeV-2), and A308-99 (HPeV-3) strains. The aligned region contains 284 amino acids. corresponding to residues 244 to 521 in the Harris polyprotein sequence (accession no. S45208). The VP3 N-terminal extension, which is specific to parechoviruses (21), is shaded. The cleavage site between VP0 and VP3 is underlined. Asterisks denote conserved residues. **Boldface** indicates HPeV reference strains.

The alignment of the predicted amino acid sequences of the partial VP0–VP3 region of Canadian HPeV-1, -2 and -3 isolates with the reference strains showed differences in the VP0/VP3 cleavage site, which was N/N in Can952219-03 and Can95224-03 (HPeV-2), N/S in Connecticut CT80-6760, T/A in Williamson and Can82047-01 (HPeV-2), N/A in Harris and all Canadian HPeV-1 isolates, and N/G in all HPeV-3 isolates (Figure 3). The length of the N-terminal extension of the VP3 protein, which is a molecular feature of parechoviruses, was also shown to differ from 27 residues in HPeV-1 of lineage I to 34 residues in HPeV-2 of lineage II. HPeV-1 of lineage II and HPeV-2 of lineage I had an extension of 28 residues, whereas an extension of 30 residues was seen in all HPeV-3 isolates (Figure 3).

### Clinical Manifestations of HPeV Infections

Twenty-eight HPeV isolates, recovered in the Quebec City area between 1985 and 2004, were available for this study (Table). This collection included 20 (71.4%) HPeV-1, 3 (10.7%) HPeV-2, and 5 (17.8%) HPeV-3. The viral isolates were recovered predominantly from NPAs (n = 16), but also from throat swab specimens (n = 2), endotracheal secretions (n = 2), stool samples (n = 7), and CSF (n = 1) (Table A1). In addition, for 2 patients, a second concomitant HPeV isolate was recovered from another site (urine in patient 7 and NPA from patient 20). Complete clinical findings were obtained for 23 (82.1%) of the 28 patients, whereas partial information was available for the remaining 5 patients.

Twenty-seven (96.4%) of the 28 patients were children <4 years (mean 11.1 months, median 7.0 months, range 1 week to 4 years). Mean age was 14.6, 6.3, and 0.7 months for HPeV-1–, HPeV-2–, and HPeV-3–infected children, respectively (p<0.001 for comparison of HPeV-1 vs HPeV-3). The 78-year-old adult excreted HPeV-1 in her endotracheal secretions. The female-to-male ratio was 1.2 (54.2% vs. 45.8%). Seventy-five percent of all HPeV isolates were recovered during the typical enterovirus season, i.e., during summer (28.6%) and fall (46.4%). The epidemiology of the newly described HPeV-3 was similar to that of other HPeVs, with 80% of this viral serotype isolated during the summer-fall period.

Among patients for whom information was available, 33.3% (8/24) had an underlying disease, including 2 children with acute lymphoblastic leukemia (ALL) (Table A1). Patients with underlying diseases represented 47.0% (8/17) of those who excreted HPeV-1 compared to none of the 7 HPeV-2–or HPeV-3–infected patients (p = 0.18 for comparison of HPeV-1 vs other HPeVs). Twenty-four (96.0%) of the 25 patients with available information were hospitalized (mean number of days 11.4, median 3.0, range 1–129). However, if the 3 patients with nosocomial HPeVs are excluded, the mean duration of hospitalization was 3.7 days; this finding was similar for the different HPeV types (mean of 3.3, 3.0, and 5.0 days for HPeV-1, -2 and -3, respectively). Only 3 (12.0%) of 25 patients were admitted to the intensive care unit. These 3 patients were infected with HPeV-1, and 2 of them (patients 6 and 26), a 4-year old boy with ALL and a 78-year-old woman, died of respiratory failure. A copathogen was found in 5 (20.8%) of 24 HPeV-infected patients, including 3 patients with human respiratory syncytial virus (HRSV), 1 with adenovirus, and 1 with human parainfluenza virus (HPIV) type 3.

Among the 24 children with available information, bronchiolitis, pneumonitis, or both, were reported as the final diagnosis in 50.0% of cases, whereas acute otitis media, sinusitis, and conjunctivitis were secondary diagnoses in 37.5%, 8.3%, and 4.2% of cases, respectively (Table A1). In addition, the final diagnosis was reported as viremia/sepsis in 29.2% of children, whereas enteritis, de novo convulsions, and shock were found in 29.2%, 4.2%, and 4.2% of patients, respectively. Final diagnosis was unknown for 3 (11.1%) of 27 children (all 3 had positive stool viral cultures). All children (7/7) with HPeV-3 and HPeV-2 infections were admitted with a diagnosis of viremia or sepsis, and all received broad-spectrum antimicrobial drugs until results of bacterial cultures (blood, CSF, urine), whereas none (0/17) of the HPeV-1-infected children had such an initial diagnosis (p = 0.001 for comparison of HPeV-1 vs. other HPeVs). Conversely, bronchiolitis was found in 8 (47.0%) of 17 children with HPeV-1 infections and in none of the patients (0/7) infected with the other 2 serotypes (p = 0.18 for comparison of HPeV-1 vs other HPeVs). However, another viral pathogen (HRSV or HPIV-3) was found in addition to HPeV-1 in 4 (50%) of 8 children with bronchiolitis.

A few cases are particularly worth noting. As previously mentioned, patient 6 was the only child who died after HPeV infection in our cohort. At the time of his HPeV-1 infection, the 4-year-old boy was receiving consolidation chemotherapy for ALL. He was admitted with high fever (40°C), dry cough, and abdominal pain. Over a course of 4 to 5 days, the following conditions developed: bilateral pneumonitis, which required mechanical ventilation, hepatitis, and septic shock complicated by renal insufficiency, for which dialysis was necessary. He died of multiorgan failure while receiving treatment with amphotericin B, acyclovir, meropenem, and vancomycin; no autopsy was performed. Apart from isolation of HPeV-1 from a throat swab, no other microorganisms (bacteria, fungi, and viruses) were recovered from blood, urine, and endotracheal secretions. Also of interest are the clinical signs and symptoms of 1-month-old twins infected by HPeV-2, who were admitted to the hospital on the same day with high fever and irritability. In 1 case (patient 20), HPeV-2 was recovered from CSF and NPA samples, whereas the virus was only isolated in NPA from the other twin (patient 21). The viruses from the 2 children had identical VP3 gene sequences. Both infants received broad-spectrum antimicrobial drugs (ampicillin plus cefotaxime) for 3 days for suspected sepsis, and both had rapid clinical improvement. Patient 26 was the only adult infected with HPeV (type 1) in our study. This 78-year-old woman was admitted to the hospital for several fractures caused by a car accident. One month after admission, bilateral pneumonia developed. She eventually died of respiratory failure 43 days after admission. An endotracheal culture was positive for HPeV-1 12 days before her death; however, results of bacterial cultures were unavailable because part of her clinical chart was destroyed a few years after her death in 1987. No autopsy report was available.

## Discussion

In this study, we retrospectively characterized 28 clinical HPeV isolates recovered during the last 2 decades in Québec, Canada, using a new RT-PCR assay. To date, our study is the only one that analyzed virologic and clinical data from the 3 HPeV serotypes. The study clearly demonstrated the importance of these pathogens and highlighted some clinical differences between infection caused by the 3 serotypes.

Virus isolation, followed by neutralization assays, a procedure that has been generally used for identification of HEVs and HPeVs, is laborious, time-consuming, and may lack sensitivity. Because of substantial sequence differences between HEVs and HPeVs, even within the conserved 5´ noncoding region (20–22), most molecular techniques designed for detection of HEVs cannot detect HPeVs (*13*,*14*). We developed a RT-PCR test for specifically detecting all 3 types of HPeV by using primers selected from the C-terminal and N-terminal regions of VP0 and VP1 HPeV capsid proteins, respectively. These regions were found to be conserved in various HPeV-1, -2, and -3 genomic sequences (data not shown). The PCR product of ≈1,000 bp encompasses the entire VP3 gene, whose sequence differs substantially between the 3 HPeV types, allowing their discrimination. By the use of this test, we were able to identify HPeV-1 and -2 reference strains as well as 28 HPeV clinical isolates, in contrast to neutralization assays that failed to recognize 2 HPeV-2 isolates by using specific HPeV-1 and -2 antisera. This molecular test appears to be specific for HPeV since no amplifications were found with samples of related viruses, including enteroviruses and rhinoviruses.

By sequencing the PCR product, we could not only differentiate the 3 HPeV types but also make an intratypic discrimination. Our phylogenetic analysis demonstrated the existence of 2 clusters for HPeV-1 strains. The first contained a Can81805-01 isolate and was closely related to the Harris reference strain; the second cluster included the remaining 19 Canadian isolates as well as recent Japanese HPeV-1 isolates (Figure 2). A similar pattern was found in a VP1-based phylogenetic study of Dutch strains in which all HPeV-1 isolates formed a cluster that was distinct from that of the Harris reference strain (13). Thus, most recent European, Japanese, and Canadian HPeV-1 isolates appear to be, to a certain extent, genetically distinct from the only known HPeV-1 reference strain. Designs of molecular or serologic diagnostic tools should take this fact into consideration. Our molecular characterization also enabled us to detect and differentiate the 2 known HPeV-2 lineages with 1 isolate close to the Williamson reference strain and the other 2 isolates being more related to the Connecticut strain. On the other hand, our HPeV-3 were found to be rather genetically homogeneous and constituted a unique cluster, which also included the Japanese HPeV-3 strains. Since HPeVs are considered important nosocomial agents, differentiating viral strains for epidemiologic purposes may be relevant. Our molecular approach demonstrated the link between 2 HPeV-2 isolates recovered from twins (100% nt identity) and showed that all other HPeV-1 and -3 isolates were unrelated.

The HPeV-1 serotype was shown to be the most frequent, representing >70% of our HPeV isolates. The HPeV-2 serotype was the less frequent, with only 3 cases reported. A very limited number of HPeV-2 infections has also been reported by a Swedish group (8). In addition, in a recent Dutch study that reported 37 HPeV isolates recovered during the 2000–2005 period, no HPeV-2 isolates were recovered compared to 27 HPeV-1 and 10 HPeV-3 (13). The recently described HPeV-3 serotype, although less frequent than HPeV-1, appears to be more frequent than HPeV-2, both in our study and in the Dutch study (13).

HPeVs have been generally isolated from stool and NPA (6–8). Accordingly, our isolates were predominantly isolated from NPA (57%) and stool (25%) specimens; however, some isolates were also recovered from other types of samples, including throat swabs, endotracheal secretions, CSF, and urine, highlighting the need to collect multiple specimens for optimal detection of these viruses.

With the exception of one 78-year-old case-patient, all HPeV infections described in this retrospective study were in children <4 years, which confirms the particular importance of these pathogens in the pediatric population (3). However, only prospective evaluation will determine the true incidence of HPeV infections in both the pediatric and adult populations. Interestingly, all of our HPeV-3 cases involved neonates with a mean age of 0.7 month, whereas HPeV-1 infections occurred in significantly older children whose mean age was 14.6 months. A similar finding was reported in Dutch patients (13), and this situation could be related to the presence of variable proportions of maternal antibodies against these different viruses.

Our study showed that HPeVs seem to share with HEVs the same epidemiologic pattern, i.e., a peak during the summer-fall months. In addition, the epidemiology of HPeV-3 appears to be similar to that of the 2 other HPeV types in our study, whereas it was slightly different in the Dutch study (13). A better knowledge of the epidemiology of these viruses may have important consequences for diagnostic laboratories. A multiplex RT-PCR that allows the simultaneous detection of HEVs and HPeVs could be particularly useful during the summer-fall seasons if the epidemiologic pattern seen in our study is confirmed by others.

Most HPeV cases reported here were associated with hospitalizations (mean hospital stay 3.7 days). No differences were found between the 3 HPeV types in this aspect. Nevertheless, only HPeV-1 infections led to severe diseases, i.e., 3 patients were transferred to an intensive care unit. Our study also showed the high rate of underlying diseases in our infected patients, which were particularly frequent (≈50%) in HPeV-1 cases. Also, substantial differences in clinical signs and symptoms were seen between the HPeV types. All 7 patients with HPeV-2 and -3 infections were admitted with a diagnosis of sepsislike illness in contrast to none of the 20 HPeV-1 patients (p = 0.001). Sepsislike illness seems to be a notable clinical feature of HPeV-3 infection, as first reported by us (11) and recently by a Dutch group (13). This clinical variation may be explained by the younger age of HPeV-2– and -3–infected children.

HPeV-1 has been reported to cause mainly gastrointestinal and respiratory diseases and fewer CNS symptoms (3,8,9,13). Accordingly, bronchiolitis, pneumonitis, acute otitis media, and enteritis were frequently diagnosed in HPeV-1–infected children in our study, whereas this HPeV type was not involved in CNS infections. Of interest, bronchiolitis was seen only in HPeV-1–infected children, representing almost 50% of the cases. However, in 4 of these 8 cases, a paramyxovirus (HRSV or HPIV) was found as copathogen; thus, the relative contribution of HPeV-1 in cases of bronchiolitis needs to be further studied. Our study also suggested that HPeV-1 could be involved in severe infections, as shown by 2 cases of bilateral pneumonia, leading to death in a 4-year-old boy with ALL and in a 78-year-old woman. Since no autopsy was performed, the role of HPeV-1 in these fatal cases cannot be confirmed, although no other microorganisms could be recovered premortem.

In conclusion, this study confirms that all 3 HPeVs are pathogens that are particularly important in the pediatric setting, that is, responsible for hospitalizations and some severe infections. HPeV infections could encompass a variety of clinical syndromes with respiratory, gastrointestinal, cerebral, and sepsislike diseases. Molecular assays now allow for their specific detection and should contribute to increased knowledge regarding their incidence, epidemiologic features, and clinical manifestations.

## Appendix

**Table A1:** Clinical findings of patients infected with human parechoviruses*

Patient/viral isolate	Sex	Age (mo)	Sample date	Sample type	HPeV type	Underlying disease	Hospital/ICU stay (d)	Diagnosis	Death	Copathogen
1/Can29218-90	F	48	Dec 3, 1990	NPA	1	Di George syndrome	3/–	Bilateral pneumonia, left AOM	No	None
2/Can40057-93	M	14	Mar 22, 1993	NPA	1	None	4/–	Pansinusitis, bilateral AOM	No	None
3/Can11750-87	M	7	May 8, 1987	Throat	1	Vater syndrome	129/–	Bronchiolitis†	No	HRSV
4/Can99190-04	F	1	Jun 27, 2004	NPA	3	None	3/–	Viremia/sepsis, left conjunctivitis	No	None
5/Can101909-04	M	15	Dec 3, 2004	NPA	1	Febrile convulsions	1/–	Febrile convulsions, left AOM	No	None
6/Can85372-02	M	48	Apr 1, 2002	Throat	1	ALL on chemotherapy	5/4	Bilateral pneumonia, septic shock	Yes	None
7/Can61165-97	F	7	Aug 20, 1997	Stool (+ urine)	1	Heart hypoplasia	13/–	Enteritis	No	None
8/Can102318-04	F	12	Dec 18, 2004	NPA	1	None	2/–	Right pneumonia, right AOM	No	None
9/Can97858-04	M	0.5	Apr 2, 2004	NPA	3	None	7/–	Viremia/sepsis	No	None
10/Can87639-02	M	18	Sep 13, 2002	NPA	1	None	2/–	Bronchiolitis, left AOM	No	HRSV
11/Can88770-02	M	9	Nov 27, 2002	NPA	1	None	2/–	Bronchiolitis, bilateral AOM	No	None
12/Can81805-01	F	18	Oct 15, 2001	Stool	1	ALL (de novo)	25/–	Enteritis,† bronchospasm†	No	None
13/Can84436-02	M	2	Feb 13, 2002	NPA	1	None	1/–	Bronchiolitis	No	HRSV
14/Can88461-02	F	5	Nov 8, 2002	NPA	1	Fallot tetralogy	5/–	Bronchiolitis	No	HPIV-3
15/Can87376-02	F	6	Aug 26, 2002	NPA	1	None	2/–	Bronchiolitis, bilateral AOM	No	None
16/Can81235-01	M	1	Sep 6, 2001	NPA	3	None	5/–	Viremia/sepsis	No	None
17/Can81554-01	F	0.75	Sep 27, 2001	NPA	3	None	5/–	Viremia/sepsis, left conjunctivitis	No	Adenovirus
18/Can82753-01	F	11	Dec 11, 2001	NPA	1	None	3/–	Bronchiolitis, bilateral AOM	No	None
19/Can82853-01	F	0.25	Dec 17, 2001	NPA	3	None	5/–	Viremia/sepsis, bilateral AOM	No	None
20‡/Can95224-03	M	1	Oct 30, 2003	CSF (+NPA)	2	None	3/–	Viremia	No	None
21‡/Can95219-03	F	1	Oct 30, 2003	NPA	2	None	3/–	Viremia	No	None
22/Can29192-90	F	16	Nov 30, 1990	Stool	1	None	3/–	Enteritis	No	None
23/Can100121-04	F	21	Aug 9, 2004	Stool	1	Asthma	0/–	Enteritis	No	None
24/Can82047-01	ND	17	Oct 30, 2001	Stool	2	ND	ND/ND	ND	ND	ND
25/Can41934-93	M	4	Jul 28, 1993	Tracheal	1	None	ND/yes (duration?)	Bilateral pneumonia, respiratory failure	ND	None
26/Can11758-87	F	78 years	Mar 25, 1987	Tracheal	1	ND	43/Yes (duration?)	Bilateral pneumonia,† respiratory failure†	Yes	ND
27/Can5188-85	ND	11	Jul 22, 1985	Stool	1	ND	ND/ND	ND	ND	ND
28/Can4541-85	ND	6	Feb 4, 1985	Stool	1	ND	ND/ND	ND	ND	ND

*HPeV, human parechovirus; ICU, intensive care unit; NPA, nasopharyngeal aspirate; AOM, acute otitis media; HRSV, human respiratory syncytial virus; ALL, acute lymphoblastic leukemia; HPIV, human parainfluenza virus; CSF, cerebrospinal fluid; ND, not determined. †Nosocomial acquisition. ‡Twins.
